# Short-term effectiveness of the national German quitline for smoking cessation: results of a randomized controlled trial

**DOI:** 10.1186/s12889-024-18104-w

**Published:** 2024-02-23

**Authors:** Simona Maspero, Simone Delle, Ludwig Kraus, Oliver Pogarell, Eva Hoch, Joachim Bachner, Kirsten Lochbühler

**Affiliations:** 1 IFT Institut für Therapieforschung, Centre for Mental Health and Addiction Research, Munich, Germany; 2 Department of Public Health Science, Centre for Social Research On Alcohol and Drugs, Stockholm University, Stockholm, Sweden; 3 Institute of Psychology, ELTE Eötvös Loránd University, Budapest, Hungary; 4 Centre of Interdisciplinary Addiction Research (ZIS), Department of Psychiatry and Psychotherapy, University Medical Center Hamburg-Eppendorf, Hamburg, Germany; 5 Department of Psychiatry and Psychotherapy, LMU University Hospital, LMU Munich, Munich, Germany; 6 Department Health and Sport Sciences, TUM School of Medicine and Health, Technical University of Munich, Munich, Germany; 7 Institute of General Practice and Family Medicine, University Hospital, LMU Munich, Munich, Germany

**Keywords:** Smoking cessation, Telephone counselling, Randomized controlled trial, Quitline, Helpline, Tobacco smoking

## Abstract

**Background:**

The objective of the present study was to examine the short-term effectiveness of the national German quitline for smoking cessation.

**Methods:**

A parallel-group, two-arm, superiority, randomized controlled trial with data collection at baseline and post-intervention (three months from baseline) was conducted. Individuals were randomized to either the intervention group, receiving up to six telephone counselling calls, or the control group, receiving an active control intervention (self-help brochure). The primary outcome was the seven-day point prevalence abstinence at post-assessment. Secondary outcomes included changes in smoking-related cognitions and coping strategies from pre- to post-assessment, the perceived effectiveness of intervention components, and the satisfaction with the intervention.

**Results:**

A total of *n* = 905 adult daily smokers were assigned to either the intervention group (*n* = 477) or the control group (*n* = 428). Intention-to-treat analyses demonstrated that individuals allocated to the telephone counselling condition were more likely to achieve seven-day point prevalence abstinence at post-assessment compared to those allocated to the self-help brochure condition (41.1% vs. 23.1%; *OR* = 2.3, 95% CI [1.7, 3.1]). Participants who received the allocated intervention in both study groups displayed significant improvements in smoking-related cognitions and coping strategies with the intervention group showing greater enhancements than the control group. This pattern was also found regarding the perceived effectiveness of intervention components and the satisfaction with the intervention.

**Conclusion:**

The present study provides first empirical evidence on the short-term effectiveness of the national German quitline for smoking cessation, highlighting its potential as an effective public health intervention to reduce the burden of disease associated with smoking.

**Trial registration:**

This study is registered in the German Clinical Trials Register (DRKS00025343). Date of registration: 2021/06/07.

## Introduction

Quitlines or telephone tobacco cessation services have been recognized as an effective public health measure [1]. Evidence suggests that telephone counselling can be a promising solution to reach a large number of the smoking population [2, 3]. Additionally, quitlines can facilitate the dissemination of evidence-based smoking cessation treatments [4, 5] by offering treatment to smokers who do not have the resources or the opportunity to access these in other settings. Overall, tobacco consumption remains one of the leading preventable contributors to both morbidity and mortality worldwide [6]. Beyond its health impact, the economic burden of tobacco-related healthcare expenses and decreased productivity is substantial [7, 8]. National quitlines might have the potential to enhance the availability and accessibility to smoking cessation treatments while maintaining treatment efficacy as well as cost-effectiveness [9–12].

Counselling by phone represents a delivery format for smokers who prefer therapist contact while alleviating barriers of physically attending a counselling service [13]. Like individual face-to-face behavioral support, telephone counselling can be tailored to the characteristics of the recipient and maximize the level of support around the needs of an individual [10, 13]. Another advantage of quitlines is that they can provide immediate cessation support [14]. Moreover, telephone counselling may be a feasible way to provide individual counselling to underserved groups, such as ethnic minorities [12, 15] or younger people [16, 17]. A recent systematic review, including 104 trials and 111,653 participants, has shown that proactive telephone counselling (calls initiated by a counsellor) for smoking cessation leads to higher rates of achieving abstinence when compared to minimal intervention controls (risk ratio (*RR*) = 1.25, 95% confidence interval (CI) [1.15 to 1.35]) [10].

Counselling services provided by quitlines have usually applied cognitive behavioral therapy (CBT) and the practice of motivational interviewing (MI) [1, 13, 18–21]. Components typically included in CBT-based smoking cessation interventions are the identification and change of maladaptive thought and behavior patterns [22, 23], the development of problem-solving and coping skills based on relapse prevention theory [18, 20], and the enhancement of self-efficacy [20, 24]. MI is a client-centered approach, that seeks to strengthen the commitment and the motivation for behavior change through the exploration and resolution of ambivalence between a person’s values and their current behaviors [19]. One study has demonstrated that CBT and MI-based quitline counselling leads to changes in psychological processes [25]. Specifically, it was found that quitline support effectively reduced positive expectations related to smoking outcomes, alleviated negative affect, enhanced self-efficacy, and increased the avoidance of external cues to smoking, while fostering the acceptance of internal cues [25].

In 2003, the World Health Organization (WHO) adopted the WHO Framework Convention of Tobacco Control (WHO FCTC) [26], providing a global response to the health and economic challenges posed by tobacco use. To assist countries in enforcing the comprehensive tobacco control policies described in the FCTC, the technical package called MPOWER was launched [27]. One of the cost-effective demand-reduction elements of the MPOWER strategy is “O – Offer help to quit tobacco use”, for example by enhancing the availability of telephone counselling services [27]. According to the Tobacco Control Scale, which demonstrates the implementation of tobacco control policies at the country level in Europe, Germany ranked among the lowest five countries in 2021 [28]. The establishment of a national quitline, however, represents one of the few proposed measures that have been implemented [28]. The Federal Centre for Health Education oversees the national quitline offering reactive telephone counselling (calls initiated by the smoker) with a maximum of five proactive follow-up calls (calls initiated by the counsellor). Despite its implementation in 1999, the counselling services of the national German Smokers Quitline have not yet been examined empirically. While the implementation of tobacco control policies is necessary, it is equally important to evaluate whether those measures are effective.

In the current study, we aimed to investigate the short-term effects of counselling services of the national German Smokers Quitline on smoking cessation. To accomplish this, we compared the abstinence rates of smokers, who received pro-active telephone counselling with those of smokers who received an active control treatment (i.e., a self-help brochure) three months from baseline (primary outcome). In addition, we investigated these effects on changes in dysfunctional smoking-related cognitions, in self-efficacy, and the acquisition of coping strategies as well as on participants’ perceived effectiveness of intervention components and on their satisfaction with the intervention (secondary outcomes). Based on previous research [10], we expected that participants in the telephone counselling condition would be more likely to achieve abstinence compared to individuals in the control condition. Given that smokers in the control condition received an active control intervention, we hypothesized that smokers in both conditions would show changes in smoking-related cognitions and coping strategies over time but that this effect would be greater for those in the intervention group. Additionally, greater perceived effectiveness of intervention components and satisfaction with the treatment were expected in the intervention group compared with the control group.

## Methods

### Study design

The present study employed a parallel-group, superiority, two-arm randomized controlled trial (RCT) design. Participants were stratified and randomly assigned to proactive telephone counselling (intervention group) or a self-help brochure (control group). Data collection took place at baseline (t_0_), as well as three (t_1_) and twelve months (t_2_) after baseline assessment. For the current analyses, data from t_0_ and t_1_ was considered.

### Sample size

To calculate the required sample size, a power analysis was carried out using the software G*Power [21]. Based on previous studies examining the effects of telephone smoking counselling and the use of self-help materials on seven-day-point-prevalence abstinence rates [10], a small effect size was expected. Assuming a significance level of 5% and statistical power of 0.8, the estimated required sample is *n* = 700 participants. The calculated sample size was corrected by a 30% dropout rate at t_2_ [7], resulting in a total sample size of *n* = 910 enrolled participants.

### Procedure

Participants were recruited by a market research company from October 2021 to July 2022 using face-to-face interviews, social media (Instagram and Facebook), and the PAYBACK panel. PAYBACK is the country’s largest customer loyalty program used by approximately half of German households. Its panel consists of over 130,000 adults, being one of Germany’s largest online access panels. Information about the study, along with a link to a digital screening survey, was provided to individuals either via email or directly through advertising. Individuals who had a minimum age of 18 years, reported having smoked at least one cigarette per day within the past 30 days, intended to quit smoking within the next four weeks, and wanted to participate in a research study were deemed eligible. A link to the baseline questionnaire (t_0_) and a personal identifier were sent to eligible individuals via email. The baseline assessment included an overview of the study and participants were asked to provide informed consent digitally. Once consent was obtained, participants completed the questionnaire on socio-demographics and smoking-related behavior.

Individuals who provided informed consent, completed the questionnaire and met the inclusion criteria once again were stratified and randomized in a 1:1 allocation ratio to one of the two study groups. Stratification was based on four criteria: (a) number of cigarettes smoked per day (1–10, 11–20, 21–30, > 30), (b) sex (female, male, non-binary), (c) age (18–30, 31–64, > 64), and (d) educational level (low, middle, high; [29]). Following the randomization procedure, participants were informed by email to which group they had been assigned and received the respective intervention no longer than two weeks after randomization. Individuals in the intervention group were contacted up to three times by the telephone counsellors to start the intervention and participants in the control group were called a maximum of five times by the study team to confirm receiving the self-help brochure. If necessary, the brochure was re-sent.

Three months after the baseline assessment, participants were invited by email to complete the post-questionnaire (t_1_). In case of non-response, the study team sent a maximum of two reminder emails and called once to encourage completion. Participants could earn up to 40€ for completing both questionnaires. For more detailed information, see the study protocol [30].

### Interventions

#### Proactive telephone counselling

The national German quitline for smoking cessation provides telephone counselling to smokers who want to quit smoking soon (i.e., in the next two weeks). The counselling protocol covers two phases of smoking cessation: preparing to quit (one session) and the maintenance of smoking cessation and relapse prevention after quitting (up to five sessions afterward). The counselling process is structured but can be tailored to individual circumstances and is based on the California Smokers’ Helpline protocol [13]. It combines MI and cognitive-behavioral approaches to help clients change their behavior [13]. The initial session (intake session) focuses on evaluating their smoking history, their smoking patterns, and their motivation to quit. The counsellor's objectives are to address any ambivalence, reinforce self-efficacy and motivation, identify challenging situations, and establish coping mechanisms. Additionally, the counsellor and the participant agree on a quit date within a 14-day timeframe. Follow-up calls are offered for maintaining smoking cessation as well as relapse prevention and take place at different intervals after the quit date. For more detailed information, see the study protocol [30].

#### Self-help brochure

Participants in the control condition received a non-tailored self-help brochure titled ‘Ja, ich werde rauchfrei!’ [‘Yes, I’ll be smoke-free!’], provided by the Federal Centre for Health Education [31]. The 92-page booklet complies with the principles of CBT and directs readers through the process of quitting smoking and maintaining abstinence [18]. It covers topics such as understanding smoking and quitting, preparing for cessation, and approaches to sustain abstinence (e.g., using coping techniques). The uptake of the brochure was self-directed. For more detailed information, see the study protocol [30].

### Measures

#### Sample characteristics at baseline

At baseline, socio-demographic data including age, gender, nationality, employment status, marital status and living situation were assessed. Additionally, information on the years of smoking, cigarettes smoked per day, cigarette dependence (FTCD; [32]), numbers of quit attempts in the past, the most recent quit attempt, the use of smoking cessation aids of former quit attempts, the importance and confidence to quit (assessed on visual analogue scales ranging from 0 (not at all important/confident) to 100 (very important/confident)) and the presence of smoking-related chronic respiratory illnesses was gathered.

#### Use of the interventions

In the intervention group, participants were asked about the number of telephone counselling calls they had received (maximum six calls). In the control group, individuals reported whether they received the self-help brochure and, if so, the extent to which they had read it. Response options ranged from “very little”, “less than half”, “more than half” to “completely” [33].

#### Seven-day point prevalence abstinence

The primary outcome was the seven-day point prevalence abstinence (yes/no) at t_1_. Individuals who had smoked cigarettes, even a single puff, or reported consuming any other type of tobacco products in the past seven days were classified as smokers.

#### Changes in smoking-related cognitions and coping strategies

The self-efficacy to refrain from smoking, positive smoking outcome expectancies, avoidance of external cues, and perceived control over withdrawal symptoms were assessed at t_0_ and t_1_.

##### Self-efficacy to refrain from smoking

Participants were presented with twelve different situations, such as “When you are with friends who smoke” and were asked to rate the level of difficulty of refraining from smoking in each situation [25, 34, 35]. A 5-point Likert scale was used ranging from 1 (very easy) to 5 (very difficult). Values were recoded so that a higher mean score indicated higher self-efficacy to refrain from smoking. Cronbach’s alpha was 0.82.

##### Positive smoking outcome expectancies

Participants were requested to indicate their degree of agreement with ten statements related to smoking, such as “Smoking helps calming down”, retrieved from the Pros of Smoking Scale [25, 36]. They provided their responses on a 5-point Likert scale, ranging from 1 (totally disagree) to 5 (totally agree). A lower mean score reflected a lower perception of smoking as being beneficial. Cronbach’s alpha was 0.76.

##### Avoidance of external cues

Participants were asked about the frequency of employing strategies to avoid external cues for smoking. Four items, such as “I remove things from my home that remind me of smoking” were used. These were rated on 5-point Likert scales ranging from 1 (never) to 5 (very often) and were derived from the stimulus control and counter-conditioning subscales of the Process of Change [25, 37]. A higher mean score indicated a greater utilization of strategies to avoid smoking-related external cues. Cronbach’s alpha was 0.75.

##### Perceived control over withdrawal symptoms

Perceived control over withdrawal symptoms was assessed using four items, such as “I believe that I am capable of dealing adequately with withdrawal symptoms from smoking” [38, 39]. Each statement was evaluated using a 5-point Likert scale, ranging from 1 (strongly disagree) to 5 (strongly agree). A higher mean score reflected a greater perception of control over withdrawal symptoms. Cronbach’s alpha was 0.91.

##### Perceived effectiveness of intervention components and satisfaction with the intervention

Participants in both study groups evaluated intervention components by indicating the helpfulness of the intervention regarding their motivation to quit/to maintain non-smoking, their management of withdrawal symptoms, their handling of strong cigarette cravings, their management of triggering situations, and their prevention of lapse/relapse [33]. Answer categories were “did not help”, “helped a little”, or “helped a lot”. Additionally, participants expressed their level of satisfaction regarding the duration of the intervention (“too short”, “about right”, or “too long”). The overall satisfaction with telephone counselling/the self-help brochure was measured using the answer categories “very unsatisfied”, “unsatisfied”, “satisfied”, or “very satisfied”. Participants were further asked if they would recommend the intervention (yes/no) and about their willingness (yes/no) to use the telephone counselling services again if needed (intervention group only) [33]. 

### Statistical analysis

Descriptive statistics (means, standard deviations and frequencies) were obtained for sample characteristics at baseline in the intention-to-treat (ITT) sample, which consisted of all randomized participants. The use of the intervention was described for individuals in the complete case (CC) sample, characterized by participants who received their allocated intervention and completed both questionnaires (t_0_ and t_1_). The ITT sample and the CC sample were compared in terms of baseline characteristics using unpaired *t*-tests and χ^2^-tests. This approach was further employed to compare the treatment groups within both sample sets.

Abstinence was analyzed for the ITT and the CC sample. According to the Russell standard, participants with missing information on their smoking status were classified as smokers [40]. The odds ratios (*OR*s) and corresponding 95% confidence intervals (*CI*s) of abstinence (vs. non-abstinence) based on group assignment were examined using binary logistic regressions. The secondary outcomes could only be analyzed for the CC sample, which consists of participants who provided those data in the t_1_ questionnaire. Mixed-repeated measurement Analyses of Variance (ANOVAs) were conducted to examine the impact of the intervention on changes in smoking-related cognitions and coping strategies. *Post-hoc* tests were performed using one-sided paired and one- (t_1_) and two-sided (t_0_) unpaired *t*-tests. To compare differences between groups in the perceived effectiveness of intervention components and the satisfaction with the intervention, χ^2^-tests were applied. The significance level was set to *p* = 0.05 for all statistical analyses. Data analyses were conducted using R version 4.3.0 [41, 42].

## Results

### Sample characteristics at baseline

**Fig. 1:**
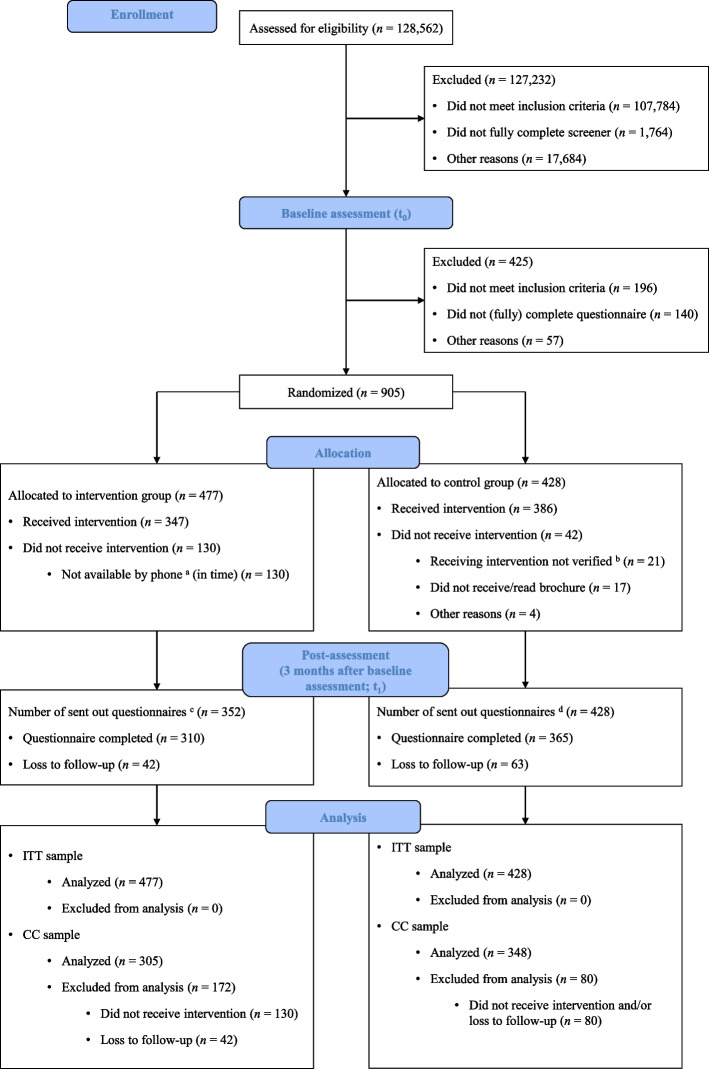
Flowchart of participants

A total of *n* = 905 participants were randomized (ITT sample). Of those, *n* = 675 completed the post-assessment (Fig. 1) and *n* = 653 received their allocated intervention (CC sample). Descriptive statistics for baseline characteristics of the ITT sample are displayed in Table 1. No significant differences were observed for any of the assessed variables between the ITT and CC samples, for both the entire sample and the treatment groups. Additionally, no significant differences were found between participants in the intervention group and the control group in both sets of samples.

**Table 1:** Sample characteristics at baseline

Baseline characteristic	Full sample (*n* = 905)	Intervention group (*n* = 477)	Control group (*n* = 428)
Age in years^a^, *M* (*SD*)	42.8 (12.7)	42.5 (12.8)	43.3 (12.6)
Gender^a^, *n* (%)			
Female	442 (48.8)	235 (49.3)	207 (48.4)
Male	462 (51.0)	241 (50.5)	221 (51.6)
Non-binary	1 (0.1)	1 (0.2)	-
German nationality^b^, *n* (%)	873 (96.5)	459 (96.2)	414 (96.7)
Education^a^^,c^, *n* (*%)*			
Low	163 (18.0)	88 (18.4)	75 (17.5)
Middle	261 (28.8)	137 (28.7)	124 (29.0)
High	481 (53.1)	252 (52.8)	229 (53.5)
Employment status, *n* (%)			
Unemployed	171 (18.9)	99 (20.8)	72 (16.8)
Casual	30 (3.3)	16 (3.4)	14 (3.3)
Part time	178 (19.7)	83 (17.4)	95 (22.2)
Full time	526 (58.1)	279 (58.5)	247 (57.7)
Marital status, *n* (%)			
Single	373 (41.2)	212 (44.4)	161 (37.6)
Married/Registered civil partnership	389 (43.0)	188 (39.4)	201 (47.0)
Divorced	132 (14.6)	72 (15.1)	60 (14.0)
Widowed	11 (1.2)	5 (1.0)	6 (1.4)
Living with a smoking partner^b^, *n* (%)	283 (31.3)	144 (30.2)	139 (32.5)
Living with other smokers in a household^b^, *n* (%)	103 (11.4)	55 (11.5)	48 (11.2)
Years of smoking, *M* (*SD*)	23.4 (12.9)	23.6 (13.3)	23.2 (12.5)
Cigarettes smoked per day^a^, *M* (*SD*)	16.2 (7.7)	16.3 (8.1)	16.1 (7.4)
Nicotine dependence^c^, *M* (*SD*)	3.9 (2.0)	4.0 (2.1)	3.8 (1.9)
Number of quit attempts in the past, *n* (%)			
None	95 (10.5)	48 (10.1)	47 (11.0)
One	93 (10.3)	41 (8.6)	52 (12.1)
Two	211 (23.3)	105 (22.0)	106 (24.8)
Three	175 (19.3)	95 (19.9)	80 (18.7)
Four	82 (9.1)	48 (10.1)	34 (7.9)
Five or more	249 (27.5)	140 (29.4)	109 (25.5)
Last quit attempt^d^, *n* (%)			
Last week	22 (2.7)	9 (2.1)	13 (3.4)
2—3 weeks ago	53 (6.5)	25 (5.8)	28 (7.3)
More than 4 weeks ago	126 (15.6)	74 (17.2)	52 (13.6)
More than half a year ago	180 (22.2)	99 (23.1)	81 (21.3)
More than a year ago	429 (53.0)	222 (51.7)	207 (54.3)
Use of smoking cessation aids in attempt to quit/stay quit^b^^,d^, *n* (%)	389 (48.0)	213 (49.7)	176 (46.2)
Importance to quit^e^, *M* (*SD*)	86.6 (19.8)	86.5 (20.5)	86.8 (18.9)
Confidence to quit^e^, *M* (*SD*)	60.8 (23.7)	60.3 (23.4)	61.3 (24.0)
Suffering from chronic respiratory illness^b^, *n* (%)	145 (16.0)	75 (15.7)	70 (16.4)

^a^Used as a stratification variable

^b^Reflects the quantity and proportion of participants responding affirmatively

^c^Assessed with the Fagerström Test for Cigarette Depencence (FTCD)

^d^Only participants with a quit attempt in the past answered the question (*n*_FullSample_ = 810,* n*_ProactiveTelephoneCounselling_  = 429, *n*_Self-helpBrochure_ = 381)

^e^Assessed on a Visual Analogue Scale with values ranging from 0 (not at all important/confident) to 100 (very important/confident)

### Attrition

To assess attrition, we conducted χ^2^-tests for categorical variables and unpaired *t*-tests for continuous variables to compare baseline characteristics between participants lost to follow- up and those who completed the questionnaire at t_1_. At t_1_, *n* = 310 participants (88.1%) completed the questionnaire in the telephone counselling group, while *n* = 365 participants (85.3%) completed the questionnaire in the control group. There was no significant difference in attrition between study groups (χ^2^(1) = 1.06, *p* = 0.303). An analysis comparing participants lost at t_1_ with those remaining on all baseline characteristics revealed a significant difference in German citizenship (χ^2^(1) = 6.06, *p* = 0.014). More participants without German citizenship were lost to follow-up compared to those with German citizenship. Additionally, participants lost at t_1_ had a significantly higher daily cigarette use at baseline (*M* = 17.5) compared to the remaining participants (*M* = 15.9, *t*(787) = 2.07, *p* = 0.039). No significant differences were observed within the study conditions on the assessed variables.

### Use of the interventions

Participants in the intervention group received on average 4.0 (*SD* = 1.6, *R* = 1 – 6) telephone counselling calls. Among individuals in the control condition, the majority read the brochure completely (56.9%) or at least half of it (31.0%). In total, 10.1% read less than half of the brochure and 2.0% read very little of the brochure.

### Seven-day point prevalence abstinence

In the ITT sample, the intervention group demonstrated a higher seven-day point prevalence abstinence rate compared to the control group with rates of 41.1% vs. 23.1%, respectively. The odds for seven-day point prevalence abstinence were significantly higher (*OR* = 2.3, 95% CI [1.7, 3.1]) for the intervention group in comparison with the control group. The CC sample yielded seven-day point prevalence abstinence rates of 63.9% in the intervention group and 28.4% in the control group. Significant higher odds (*OR* = 4.5, 95% CI [3.2, 6.2]) for seven-day point prevalence abstinence were found for the intervention group compared to the control group.

### Sensitivity analyses

To determine the robustness of the findings, abstinence was additionally defined as no use of any tobacco products or alternative products [43]. Moreover, we accounted for randomization strata and calculated adjusted and unadjusted *OR*s for abstinence. The findings remained consistent when considering abstinence from alternative products as part of the outcome or adjusting for randomization strata.

### Changes in smoking-related cognitions and coping strategies

There was a significant group × time interaction effect on self-efficacy to refrain from smoking (*F*(1, 651) = 54.32, *p* < 0.001), positive smoking outcome expectancies (*F*(1, 651) = 23.52, *p* < 0.001), avoidance of external cues (*F*(1, 651) = 10.74, *p* = 0.001) and perceived control over withdrawal symptoms (*F*(1, 651) = 40.44, *p* < 0.001). No significant inter-group differences were found at baseline regarding the self-efficacy to refrain from smoking (*t*(651) = -0.48, *p* = 1.00), positive smoking outcome expectancies (*t*(651) = -0.41, *p* = 1.00), avoidance of external cues (*t*(651) = -1.46, *p* = 0.291) and perceived control over withdrawal symptoms (*t*(651) = 0.59, *p* = 1.00). At post-assessment, the intervention group demonstrated significantly greater self-efficacy to refrain from smoking (*t*(651) = 6.57, *p* < 0.001), lower positive smoking outcome expectancies (*t*(651) = 4.23, *p* < 0.001), higher avoidance of external cues (*t*(651) = -4.98, *p* < 0.001) and higher perceived control over withdrawal symptoms (*t*(651) = -6.05, *p* < 0.001) compared to the control group. Significant intra-group differences from t_0_ to t_1_ were found in all the assessed psychological constructs for both groups (Table 2).

**Table 2:** Means, standard deviations, and t-test statistics for changes in smoking-related cognitions and coping strategies

Smoking-related cognitions and coping strategies	Intervention group (*n* = 305)						Control group (*n* = 348)					
	Baseline		Post-assessment		*t*(304)	*p*	Baseline		Post-assessment		*t*(347)	*p*
	*M*	*SD*	*M*	*SD*			*M*	*SD*	*M*	*SD*		
Self-efficacy to refrain from smoking^a^	2.3	0.6	3.4	1.0	-19.14	< .001	2.4	0.6	2.9	0.8	-14.69	< .001
Positive smoking outcome expectancies^a^	2.8	0.5	2.3	0.6	12.96	< .001	2.8	0.5	2.5	0.5	9.32	< .001
Avoidance of external cues^a^	2.7	0.8	3.5	0.9	-11.60	< .001	2.6	0.9	3.1	0.9	-9.46	< .001
Perceived control over withdrawal symptoms^a^	3.2	0.9	3.9	1.0	-11.14	< .001	3.3	0.9	3.5	1.0	-3.59	< .001

^a^*R* = 1–5

### Perceived effectiveness of intervention components and satisfaction with the intervention

Compared with the control group, participants in the intervention group reported significantly higher perceived effectiveness of the intervention components: their motivation to quit, the management of their withdrawal symptoms, strong cravings, situations that trigger craving, and the prevention of lapse/relapse (Table 3).

**Table 3:** Frequencies and chi-square results for the perceived effectiveness of intervention components

Intervention component	Intervention group (*n* = 305)		Control group (*n* = 348)		χ^2^		
	*n*	%	*n*	%	Value	*df*	*p*
Motivation to quit smoking/maintain non-smoking					50.09	2	< .001
Did not help	31	10.2	67	19.3			
Helped a little	123	40.3	200	57.5			
Helped a lot	151	49.5	81	23.3			
Management of withdrawal symptoms					26.11	2	< .001
Did not help	61	20.0	124	35.6			
Helped a little	154	50.5	166	47.7			
Helped a lot	90	29.5	58	16.7			
Management of strong cravings for cigarettes					23.67	2	< .001
Did not help	71	23.3	118	33.9			
Helped a little	145	47.5	180	51.7			
Helped a lot	89	29.2	50	14.4			
Management of situations that trigger strong cravings for cigarettes					28.51	2	< .001
Did not help	70	23.0	112	32.2			
Helped a little	126	41.3	175	50.3			
Helped a lot	109	35.7	61	17.5			
Prevention of lapse or relapse					58.54	2	< .001
Did not help	74	24.3	156	44.8			
Helped a little	120	39.3	148	42.5			
Helped a lot	111	36.4	44	12.6			

Individuals in the intervention group expressed significantly higher satisfaction regarding both the intervention’s duration and the overall intervention when compared to those in the control group (Table 4).

**Table 4:** Frequencies and chi-square results for the satisfaction with the intervention

	Intervention group		Control group		χ^2^		
	(*n* = 305)		(*n* = 348)				
	*n*	%	*n*	%	Value	*df*	*p*
Satisfaction with the length of the intervention, %					24.60	2	< .001
Too short	16	5.2	37	10.6			
About right	269	88.2	253	72.7			
Too long	20	6.6	58	16.7			
Overall satisfaction with the intervention, %					54.58	3	< .001
Very unsatisfied	27	8.9	16	4.6			
Unsatisfied	27	8.9	50	14.4			
Satisfied	133	43.6	227	65.2			
Very satisfied	118	38.7	55	15.8			

No significant differences between groups were found regarding the participants’ recommendation of the intervention to others (intervention group = 84.9%, control group = 80.7%, χ^2^(1) = 1.70, *p* = 0.193). Additionally, 76.4% of individuals in the intervention group expressed their willingness to contact the quitline again if needed.

## Discussion

The present study examined the short-term effectiveness of the national German quitline for smoking cessation using a two-arm RCT comparing the counselling services to a self-help brochure. As expected, proactive telephone counselling outperformed the support of a self-help brochure, with a greater likelihood of seven-day point prevalence abstinence three months from baseline. The telephone counselling's superiority may be attributed to its ability to provide personalized support tailored to an individual's specific requirements [10, 13] in contrast to a non-tailored self-help brochure. Moreover, although the self-help brochure also followed CBT principles, CBT has been demonstrated to yield more substantial effects when supplemented with guidance, for example from a counsellor [44, 45]. Lastly, a comprehensive approach that combines elements of CBT and MI has been shown to be more effective in supporting smoking cessation compared to solely using CBT [46].

The current result aligns with prior research, indicating that proactive telephone counselling for smoking cessation yields greater abstinence rates when compared to minimal intervention controls [10]. However, the present smoking cessation rates in both conditions were higher than those in previous studies [10, 21, 47]. For example, the European Smoking Cessation Helplines Evaluation study (ESCHER) focused on evaluating national quitline usage and its impact on smoking cessation rates across multiple European countries [21]. Across countries, an overall seven-day point prevalence abstinence rate of 14.3% was reported among individuals who were preparing to quit and received quitline counselling. Regarding the effectiveness of printed self-help resources for smoking cessation, a meta-analysis demonstrated abstinence rates ranging from 2 to 10% [47].

The disparity in cessation rates in comparison with previous studies might be explained by the time-point of abstinence assessment and the included samples. Compared to the three-month post-assessment in the current study, previous studies [10, 21, 47] evaluated abstinence at least six months after the intervention. Although relapses are most common within the first five to ten days of a quit attempt, they are still likely to occur in the following months [48]. Abstinence rates for both groups in the present study are therefore expected to decrease over time. The difference in abstinence rates between the current study and previous research might also be attributed to differences in the study samples. In the current study, participants were eligible to participate if they intended to quit smoking within the next four weeks. This eligibility criterion was less strict in prior research [10]. However, the intention to quit smoking was identified as a predictor of quit attempts [49, 50], possibly explaining the higher abstinence rates in the current study.

As expected, both study groups demonstrated significant enhancements in smoking-related cognitions and coping strategies. Nevertheless, individuals who received quitline counselling reported greater self-efficacy to refrain from smoking, less positive smoking outcome expectancies, higher avoidance of external cues and higher perceived control over withdrawal symptoms than participants who read the self-help brochure. Those greater enhancements in the telephone group may be attributed to the structured, individually tailored counselling using evidence-based elements of CBT and MI targeting those psychological processes. However, the content of the brochure was also based on elements of CBT, possibly explaining the found improvements in the control condition over time. By including CBT-based strategies like cognitive reorganization to target unhelpful thought patterns, the content of the brochure intended to target the same psychological processes.

The same pattern of results was found regarding the participants’ perceived effectiveness of intervention components and their satisfaction with the intervention. In both groups, more than half of the participants perceived the intervention components as at least a little helpful with regard to their motivation to quit, their ability to manage withdrawal symptoms, their ability to manage strong cravings for cigarettes and situations that trigger strong cravings, as well as with their ability to prevent lapses or relapses. However, the individuals in the telephone counselling condition perceived the intervention as more helpful and were more satisfied with the intervention than the participants in the control condition.

This study holds significant implications. Of German smokers making an annual attempt to quit smoking (19.9%), only 13.0% use evidence-based strategies [51]. While methods like brief consultations with physicians and over-the-counter nicotine replacement therapy are used most often (5.3% and 4.9%, respectively), merely 0.8% of individuals turn to quitlines for support [51]. Given the short-term effectiveness of the German national quitline for smoking cessation and its potential for long-term effectiveness, future research should investigate the factors that contribute to its underutilization.

Literature suggests that barriers to smoking cessation treatments possibly include the cost of treatment, a lack of awareness regarding the availability of cessation support and a reduced interest in conventional approaches [52–54]. As the national German quitline for smoking cessation offers telephone counselling free of charge, the cost of treatment does not constitute a barrier in Germany. However, future research needs to examine smokers’ awareness of the existence of the national quitline and its services and possibly increase its promotion or adapt promotion strategies.

A reduced interest in conventional smoking cessation interventions and a personal preference for a certain type of intervention emphasize that telephone counselling should supplement other forms of smoking cessation counselling. The results of the current study confirm that quitline counselling should be an important part of public health provision [20]. While quitlines may produce lower abstinence rates compared to clinical interventions, as a public health approach, they can cast a wider net to reach a larger number of smokers [20, 55] and, therefore, may have a greater potential to reduce rates of morbidity and mortality [56]. Considering that evidence-based treatments provided through national quitlines have been well-established in alternative formats, such as face-to-face individual or group therapy for smoking cessation [20, 57, 58], quitline counselling should be one element in a comprehensive stepped-care strategy of smoking cessation support. To accommodate the diverse needs and preferences of smokers, a range of support options should be available, offering varying levels of intensity and degrees of anonymity.

The results presented are subject to limitations. First, the present sample differs from callers to the national German quitline for smoking cessation and recipients of pro-active follow-up calls with regard to socio-demographic and smoking-related variables [59]. Consequently, the external validity of the results is somewhat limited. Although this could have been accounted for by including only individuals in the study who called the quitline, randomizing callers to the control condition would have been ethically inappropriate. Second, using self-reported data to assess abstinence may have led to an overestimation of actual abstinence rates [60]. Nevertheless, biochemical validation in smoking cessation research may not be necessary and the misrepresentation of the smoking status is considered rare [61]. Third, the current study examined the short-term effectiveness of telephone counselling services. However, evidence on the long-term effectiveness is needed to ascertain sustainability of the treatment effects [40]. Results on whether the counselling services of the national German quitline for smoking cessation leads to long-term abstinence will be published once data analysis is completed.

This study demonstrates that the national German quitline for smoking cessation is effective in the short term. In addition to the high abstinence rates, the positive changes in intervention targets and the participants’ satisfaction with the telephone counselling highlights the potential of the provided services to curb smoking addiction and reduce the associated burden of disease. The present findings carry implications for public health policy, indicating that the availability and the promotion of telephone counselling services could serve as a valuable strategy for augmenting smoking cessation initiatives in Germany.

## Data Availability

Anonymized study data and statistical codes to analyses may be made available on request from the corresponding author following study closure.

## Abbreviations

ANOVA: Analysis of Variance
BZgA: Bundeszentrale für gesundheitliche Aufklärung, Federal Centre for Health Education
CBT: Cognitive behavioral therapy
CC: Complete case
CI: Confidence interval
CONSORT: Consolidated Standards for Reporting Trials
DGP: Deutsche Gesellschaft für Psychologie, German Psychological Society
DRKS: Deutsches Register klinischer Studien
FCTC: Framework Convention on Tobacco Control
FTCD: Fagerström Test for Cigarette Dependence
IFT: Institut für Therapieforschung
ITT: Intention-to-treat
MI: Motivational interviewing
RCT: Randomized controlled trial
RR: Risk ratio
WHO: World Health Organization
